# Application and configuration analysis of electric muck transfer equipment in plateau railway tunnel: a case study in southwest China

**DOI:** 10.1038/s41598-024-57628-4

**Published:** 2024-03-27

**Authors:** Xiaoxu Yang, Yuming Liu, Kai Liu, Jianying Wei, Guangzhong Hu, Shifan Pei

**Affiliations:** School of Economics and Management, Beijing Jiaotong University, Beijing, 100044 China

**Keywords:** Application and configuration analysis, Electric equipment, Muck transfer, Drilling and blasting method, Plateau railway tunnel, Environmental sciences, Environmental social sciences

## Abstract

The burgeoning development of railway construction in plateau regions of southwest China necessitates innovative and environmentally sustainable approaches, particularly in the realm of tunnel construction, where the transfer of muck poses significant operational and environmental challenges. This research, pivoting around the application and configuration of electric muck transfer equipment in plateau railway tunnels, seeks to dissect the potentialities and impediments of transitioning from conventional diesel-powered machinery to electric alternatives, with a spotlight on mitigating environmental impacts and enhancing operational efficiency. Through an analytical lens, the study employs a case study methodology, leveraging data and insights from existing electric equipment models and their applications, provided by major manufacturers in China, to weave a comprehensive narrative around the practicalities, specifications, and challenges embedded in the adoption of electric machinery in plateau environments. The findings unveil a nuanced landscape, where the environmental and operational advantages of electric equipment are juxtaposed against a backdrop of technological, financial, and infrastructural hurdles, thereby crafting a complex tapestry of opportunities and challenges. The research further extrapolates policy recommendations and practical guidelines, advocating for a harmonized amalgamation of governmental policies, technological advancements, and strategic planning to navigate through the identified challenges and optimize the integration of electric equipment in tunnel construction practices. Envisaging future research pathways, the study underscores the criticality of perpetuating technological innovations, policy adaptations, and interdisciplinary research to further refine and enhance the application of electric muck transfer equipment in plateau railway tunnel projects, thereby contributing to the broader narrative of sustainable construction practices in challenging terrains.

## Introduction

Railways, often regarded as the lifeblood of China's national economy, serve as crucial infrastructure and play a pivotal role in major livelihood projects. Their significance in shaping China's socio-economic landscape cannot be overstated^1–4^. Over the years, the methodologies employed in railway tunnel construction in China have witnessed a transformative evolution, culminating in a robust and mature technical system. A landmark in this journey was the construction of the Dayaoshan Tunnel of the Hengyang Guangzhou Double Track Railway in the 1980s. This era marked a paradigm shift from traditional manual tunneling methods, reminiscent of the "one man, one pick" approach, to a more sophisticated mechanized construction mode^5^. This transition paved the way for China to delve into innovative construction methods, blending drilling, blasting, and mechanized techniques, thereby enhancing the efficiency, quality, and safety of tunnel construction^6–11^.

However, as China's infrastructural ambitions expanded into its remote and challenging mountainous terrains, the challenges multiplied. The surge in high-altitude railway tunnels and blasting projects brought to light the limitations of conventional diesel-powered mechanical equipment. Their diminished dynamic and economic performance, leading to significant power losses, rendered them inadequate for high-standard construction, posing substantial technical challenges for mechanized drilling and blasting operations.

The essence of efficient tunnel construction lies in the judicious selection and configuration of machinery. Over the years, numerous scholars have ventured into this domain, aiming to devise optimal equipment configurations for plateau railway tunneling. Their endeavors have been directed towards harnessing established equipment systems and fine-tuning selection strategies in light of available technological advancements^12–15^. For instance, Xiang and Yuan's pioneering work laid down a comprehensive mechanical equipment coordination and operational blueprint tailored for large-scale passenger-only line tunnels, encompassing diverse processes from excavation to lining^16^. Song and Xia's innovative carbon emission model, rooted in life-cycle assessment and the carbon emission factor method, offers a nuanced perspective on the environmental footprint of tunneling mechanisms^17^. Sun et al.'s insights into tunnel slag discharge techniques, especially their proposal of a continuous band slag discharge method, underscore the potential benefits of modern slag extraction systems, especially in challenging terrains like frozen soils and plateaus^18^.

While past research has predominantly focused on optimizing conventional fuel oil equipment configurations or dissecting specific technologies tailored for plateau environments, there remains a conspicuous void in systematic research on electric construction equipment, especially in highly mechanized configurations^19^. Electric equipment, with its low energy costs, reduced maintenance overheads, and zero-emission capabilities, offers a promising alternative. Its advantages are particularly pronounced in plateau regions, where climatic constraints amplify the benefits of electric appliances in railway tunnels^20^. Furthermore, given the significant time and machinery resources consumed by tunneling muck transfer operations, coupled with their environmental implications, the modern development paradigm necessitates innovative approaches in the application and configuration of electric equipment for muck transfer in plateau railway tunnels.

## Background

### Requirements of sustainable development concept

In the wake of China's commitment to peak its carbon emissions before 2030 and achieve carbon neutrality before 2060, known as the 'Dual Carbon' targets^21,22^, the nation has been steering its industries towards more sustainable practices. The Ministry of Industry and Information Technology, in 2020, initiated an action plan to expedite the electrification of public sector vehicles, including construction machinery and heavy trucks, thereby aligning with the national agenda to mitigate carbon emissions. Table 1 encapsulates a series of policies and regulations, reflecting China's strategic approach towards embracing green and low-carbon technologies across various sectors, including transport and manufacturing. These policies not only underscore the importance of adopting new energy and clean energy vehicles but also emphasize enhancing charging infrastructure and promoting green products and technologies^23^.

**Table 1:** Summary of China's recent policies and regulations.

Time	Publishing department	Policy name	Specifics
December 2022	National Development and Reform Commission	Implementation Plan for the 14th Five-Year Plan to Expand Domestic Demand	Actively develop the green and low-carbon consumption market, and encourage e-commerce platforms to expand sales of green products. Establish and improve standards, identification and certification systems for green products, and carry out evaluation of green products. Vigorously promote new-energy vehicles and new-energy and clean-energy vessels
October 2022	Ministry of Transport	Opinions on the Comprehensive and In-depth Promotion of Green Transport Development	Promote the professional standardization of transport equipment, promote the application of new energy and clean energy vehicles and vessels, including tasks such as promoting the standardization of ship types, eliminating old vessels, encouraging the application of new energy vehicles, and improving charging facilities on the road network and refueling facilities on high-grade inland waterways
May 2022	National Development and Reform Commission, National Energy Administration	Implementation Plan on Promoting the High Quality Development of New Energy in the New Era	Guiding the whole society to consume new energy and other green electricity, and encouraging all kinds of users to buy products made of green power such as new energy
January 2022	National Development and Reform Commission, National Energy Administration	Opinions on Improving Institutional Mechanisms and Policy Measures for Green and Low Carbon Energy Transition	Promote the green and low-carbon transformation of transport, optimize the transportation structure and implement green and low-carbon transport facilities and equipment. Support the layout and construction of transport energy supply stations in terms of land and space, carry out the construction of multi-energy integrated transport energy supply stations, promote pilot demonstrations of energy interaction between new energy vehicles and power grids, and promote the synergistic development of vehicle-pile and ship-shore
November 2021	Ministry of Transport	The 14th Five-Year Plan for the Development of Integrated Transport Services	The Government will upgrade the level of green travel equipment, promote the large-scale application of new energy vehicles, accelerate the construction of charging infrastructure, and carry out "endurance projects" of green travel. Vigorously cultivate a green travel culture, improve public participation mechanisms, and build a green travel service system that is reasonably laid out, ecologically friendly, clean and low-carbon, intensive and efficient
August 2021	State Council	Notice on the Management Measures for the Gradual Utilization of New Energy Vehicle Power Battery	Strengthen the management of cascade utilization of power batteries for new energy vehicles, improve the level of comprehensive utilization of resources, ensure the number of battery products for cascade utilization, and protect the ecological environment
July 2020	Ministry of Industry and Information Technology	Decision of the Ministry of Industry and Information Technology on Amending the Regulations on the Administration of Access to New Energy Vehicle Manufacturing Enterprises and Products	The Ministry of Industry and Information Technology is responsible for the implementation of national access and supervision of new energy vehicle production enterprises and products
March 2019	Ministry of Industry and Information Technology	/	According to GB 17,691–2018 Emission Limits and Measurement Methods of Pollutants from Heavy Diesel Vehicles (National VI), heavy-duty gas vehicle products will implement the emission requirements of Phase A of National VI on July 1st, 2019

The transition to electric equipment in construction, especially in tunneling operations, is further necessitated by the changing demographics of the Chinese labor force^24^. With an aging population and increasing labor costs, there is a growing need for more efficient, less labor-intensive construction methods. Electric muck transfer equipment offers a solution to these challenges, providing a cleaner, safer, and more sustainable alternative to traditional diesel-powered machinery^25^. While acknowledging the significant strides made in electric vehicle technology and infrastructure, it's crucial to place electric construction equipment within a context that considers multiple alternatives, including hybrid and hydrogen fuel cells. This study examines electric equipment's viability against such alternatives, considering factors like energy efficiency, environmental impact, and adaptability to plateau conditions.

### Plateau climate and environmental limitations

Tunnel construction, particularly in plateau regions, is inherently energy and resource-intensive, leading to significant greenhouse gas emissions^26,27^. A primary source of these emissions is the diesel-powered machinery commonly used in drilling and blasting methods, such as excavators, loaders, and dump trucks. These machines, while efficient at lower altitudes, face operational challenges in plateau environments due to the thin air and low oxygen levels.

At lower altitudes, the abundance of oxygen mitigates the concentration of harmful gases emitted by construction machinery, resulting in relatively lower pollution levels. However, in plateau regions, the situation is markedly different (see Table 2). The reduced oxygen content in the air leads to inefficient combustion in diesel engines, significantly increasing the emission of hazardous gases such as nitrogen oxides, oxygenated hydrocarbons, and particulates^28,29^. This not only poses serious health risks to the construction workforce but also complicates the ventilation requirements in tunnel construction, impacting overall efficiency and safety.

**Table 2:** Changes of main performance parameters of diesel engine with altitude^30^.

Altitude/m	Atmospheric pressure/kPa	Ambient temperature variation	Non-supercharged diesel engine				Supercharged diesel engine		
			Boiling point of water/℃	Power dip/%	Increased fuel consumption/%	Exhaust temperature rise/%	Power dip/%	Increased fuel consumption/%	Exhaust temperature rise/%
2000	79.2	For every 1000 m rise in altitude, the ambient temperature drops by about 6.5 degrees Celsius	93.7	18	12	20	2	6	10
3000	70.1		90.2	27	18	30	3	9	15
4000	61.6		85.8	36	24	40	5	12	20
5000	54.0		82.7				10	15	25

**Figure 1:**
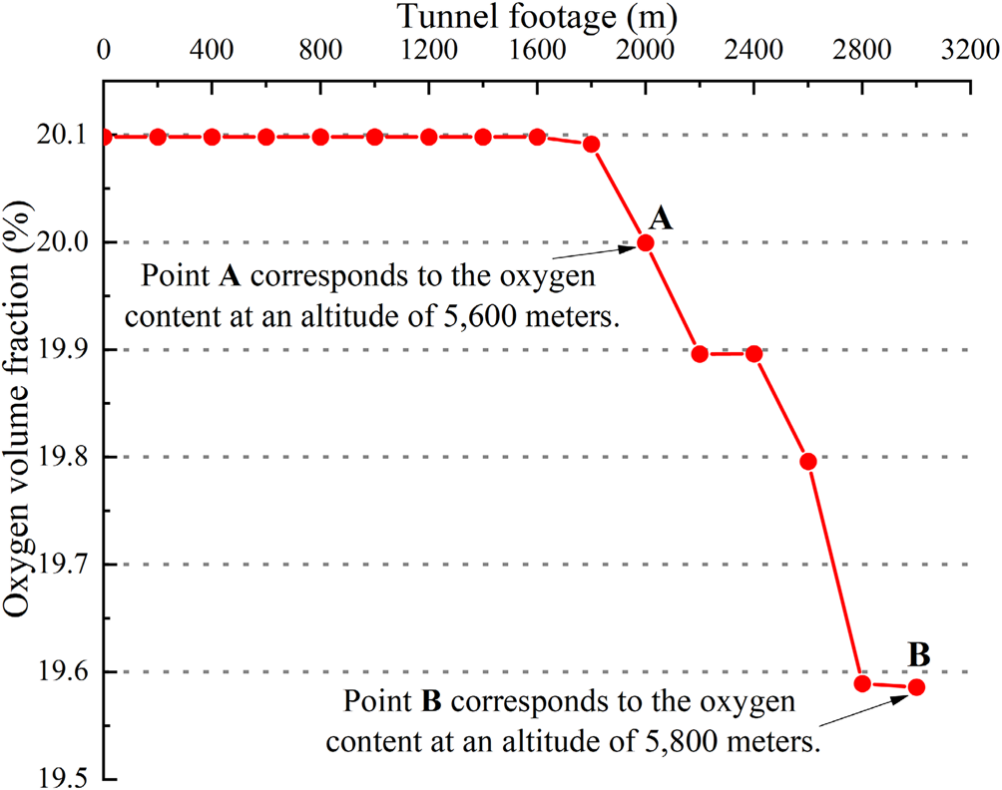
Sketch of variation of oxygen content in high-altitude tunnel with footage depth^31^.

At the same time, the problem of oil machinery and equipment competing for oxygen with front-line workers in the plateau area also cannot be ignored, due to the continuous oxygen consumption of construction personnel and mechanical equipment in the tunnel, the oxygen content in the tunnel at high altitude will gradually decrease with the increase of tunnel footage, as shown in Fig. 1. Excessive hypoxia can lead to physical discomfort, fainting, and even death for workers^32,33^, adding to the strain of long-distance ventilation in the plateau tunnels.

Currently, electric equipment, primarily powered by lithium iron phosphate batteries, offers a sustainable alternative. By substituting internal combustion engines with electric motors, electric equipment reduces power losses unaffected by air pressure and oxygen fraction changes^34,36^. This advantage is particularly significant in tunnels above 3000 m altitude, where electric muck removal equipment surpasses traditional diesel-powered machinery in operational efficiency. Electric drive systems do not consume oxygen during operation, and with zero exhaust emissions, they address the issue of competing for oxygen with workers, alleviating ventilation and oxygenation pressures in high-altitude tunnel construction^35,37^. Moreover, field data indicate that electric equipment generates significantly lower external noise levels, 20–30 dB lower than comparable diesel machinery, effectively improving the work environment.

The plateau climate presents unique challenges that exacerbate the limitations of diesel-powered equipment. Electric equipment, with its inherent advantages in energy efficiency and lower emissions, offers a compelling alternative. This study aims to fill the research void by systematically comparing the performance, environmental impact, and practicality of electric versus diesel equipment in these demanding environments.

## Equipment research and development status

### Electric muck transfer equipment

The vigorous development of electric vehicles has led to the development of power batteries and electric motors, and has also made it possible to electrify construction machinery. Compared with electric vehicles, electric construction machinery and equipment is still in its infancy^36,37^. Currently, tunnel equipment can be divided into three categories according to their power source: (1) Pure electric equipment: common in secondary lining equipment, such as invert trestle, waterproof board trolley, lining trolley, etc. Such equipment have no requirements on the oxygen content of the air in which they operate and do not pollute the tunnel environment. (2) Diesel-electric hybrid equipment: widely used in advance geological prediction, excavation operation, initial lining, the use of diesel power walking, electric power work. The walking time of such equipment takes up a small proportion compared to the working time, which has a slight effect on the pollution and oxygen consumption in the tunnel. (3) Pure diesel power equipment: common in muck transfer equipment, such as excavators, loaders, dumpers, etc. Such equipment is extra sensitive to oxygen and pressure changes in the working environment, and will produce additional toxic gas due to insufficient fuel combustion, causing serious pollution to the tunnel. In addition, the muck transfer line is still dominated by purely diesel power equipment. Muck transfer operations accounted for approximately half of the total cycle time throughout the drilling and blasting method. Therefore, plateau railway tunnel construction for electric equipment demand, mainly concentrated in muck transfer lines.

However, there is no complete set of electric muck transfer equipment for sale in China, and the research and development of related enterprises is concentrated in the small and medium-sized stage, which cannot cover all the working conditions of the plateau railway for the time being. According to the current status of electric equipment products of major manufacturers in China (see Table 3), it can be found that compared with traditional diesel power equipment, the specifications and models of electric excavators and loaders are relatively simple, while the specifications and models of electric dump trucks are relatively various.

**Table 3:** Electric equipment models that have been put into production by major Chinese manufacturers.

Name of manufacturer	Main products	Product model	Specification	Recharging mode
SANY HEAVY INDUSTRY CO., LTD	Electric excavator	SY19E, SY215E	1.9t-Class, 21.5t-Class	Recharge mode
	Electric dump truck	SYM3312ZZX3BEV ,SYM3251ZZX3BEV, SYM3312ZZX2BEV	25t-Class, 30t-Class	Recharge mode, Battery-swap mode
XUZHOU CONSTRUCTION MACHINERY CO., LTD	Electric excavator	XE35E, XE215E, XE270E	3.5t-Class, 23.5t-Class, 27.5t-Class	Recharge mode, Battery-swap mode
	Electric loader	XC958-EV	5t-Class	Recharge mode
	Electric dump truck	E300-series, E500-series, E700-series, P600-series, P900-series,	8t-Class, 18t-Class, 25t-Class, 30 t-Class, 50t-Class, 90t-Class	Recharge mode, Battery-swap mode
SINOMACH CHANGLIN CO., LTD	Electric excavator	323E	23t-Class	Recharge mode
	Electric loader	950E, 955E	5t-Class	Recharge mode
GUANGXI LIUGONG MACHINERY CO., LTD	Electric excavator	924F-E/924F-ETN	24.9t-Class	Recharge mode
	Electric loader	856E-MAX	5.5t-Class	Recharge mode
SUNWARD INTELLIGENT EQUIPMENT GROUP	Electric excavator	SWE240FED	24t-Class	Recharge mode

### Charging infrastructure

1. Charging pile

Charging infrastructure is an essential supporting guarantee for the efficiency of electric equipment. Currently, the construction technique of fixed charging piles is relatively mature. It is feasible to construct centralized charging stations for electric equipment outside the tunnels and configure high-power double-gun quick charging piles. China has also formulated a series of national, industrial and related corporate standards and norms for charging technology. In the short term, shortening charging times and improving endurance are undoubtedly the technological priorities for the market.

**Figure 2:**
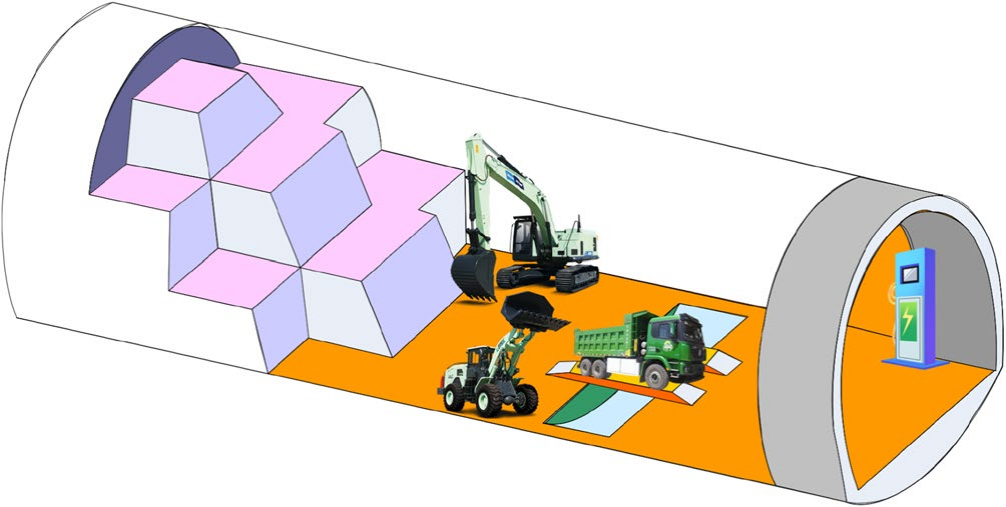
Layout of charging piles in the secondary lining area.

The charging pile has a low construction cost and can be distributed flexibly according to the distribution of vehicles. At the same time, one charging pile can satisfy 3–4 vehicles for a whole day's operation. Depending on the operation conditions of the vehicles at the project site, energy can be replenished by exploiting the waiting time for loading and unloading materials and the rest time of the drivers. Equipped with high-power charging piles, the energy can be replenished by 40–50% in half an hour and the driving endurance of vehicles can be increased by 50-70 km. When the tunneling distance is not lengthy, the charging pile can be fixed outside the tunnel. As the tunneling distance increases, it can be considered to fix the charging pile in the secondary lining area of the tunnel and push forward with the tunneling (see Fig. 2). However, it is necessary to further verify the reliability of the supporting charging piles in the humid, high temperature and relatively muddy environment of the tunnel and how the structural firmness meets the construction requirements under frequent movement.

(2) Battery swap station

**Figure 3:**
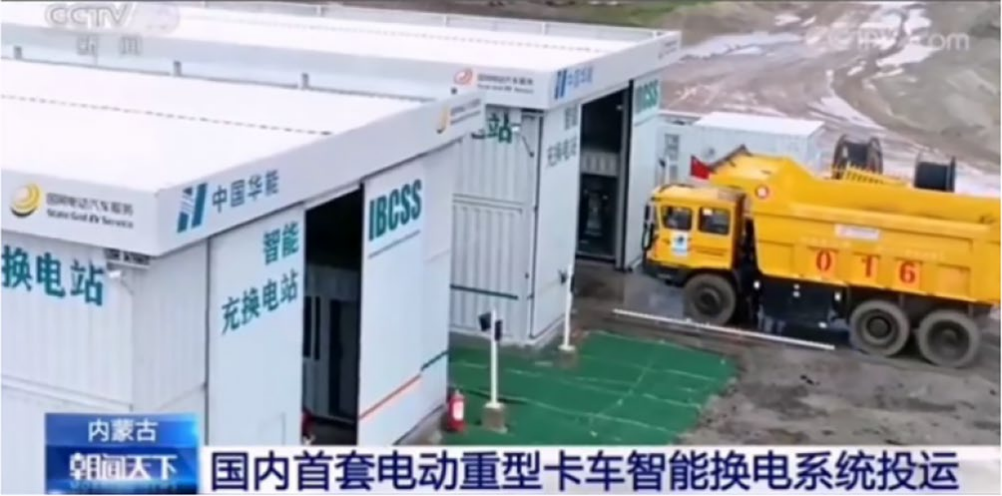
The first intelligent electrical change system for electric heavy truck in China.

Compared with charging facilities, the construction of electricity exchange facilities is relatively in the early stages of development. In May 2020, the power station was included in the Government Work Report for the first time, as an vital part of the improved infrastructure. In July 2020, the Ministry of Industry and Information Technology made it clear that it supports electric switching services and encourages auto companies to develop electric switching models. Later that year, at the Huaneng Yimin coal and electricity open-pit mine, China's first set of electric heavy-duty trucks with intelligent power shift systems went through a 100-day high-intensity trial operation, which started the process of electric energy replacement for China's large construction vehicles (see Fig. 3).

The one-time investment cost of the battery swap station is relatively costly (about five to six million CNY), and it covers a large area (about 150–200 square meters), which requires centralized operation. When the number of battery-swap mode machines exceeds 50, the size economy effect can be generated. The electric-changing mode can be considered in the operation scenarios that require high charging efficiency.

## Application and configuration analysis

### Project overview

As a new product, electric muck transfer equipment is rarely used in the engineering construction sector, and is still in the trial phase in the railway tunnel construction sector^38,39^. The project under study is located in a challenging terrain in the plateau mountainous region of southwest China. This tunnel, a crucial part of the railway network expansion in the region, spans approximately 20 km in length. It features a maximum buried depth exceeding 1000 m and is situated at an average elevation of over 3500 m. These geographical and environmental conditions present unique challenges, including significant variations in temperature, reduced oxygen levels, and complex geological structures.

In this project, electric muck transfer equipment, a relatively new innovation in railway tunnel construction, was employed. The equipment, sourced from mainstream manufacturers, matches the traditional diesel-powered equipment in specifications, performance, safety, and reliability. The construction enterprise adopted electric excavators, loaders, dump trucks, and supporting charging infrastructure, creating a comprehensive demonstration of electric muck transfer in tunnel construction.

The operational cycle of the project typically involves three construction phases every two days. Each phase includes a muck transfer duration of approximately three hours, handling about 260 cubic meters of muck. This translates to an average daily working time of 4.5 h for the muck transfer equipment, with an average daily muck transfer volume of 390 cubic meters, or an hourly rate of 86.67 m^3^/h.

Preliminary observations indicate that the performance of the electric equipment aligns closely with that of traditional diesel-powered machinery. For the purpose of a subsequent economic analysis, we have made certain assumptions: excavators and loaders are considered over a depreciation period of ten years, dump trucks over five years, and battery life is also estimated at five years. These calculations do not take into account the salvage value of the equipment. These parameters are set to construct a framework for a detailed economic analysis, which will be elaborated in the following sections.

**Figure 4:**
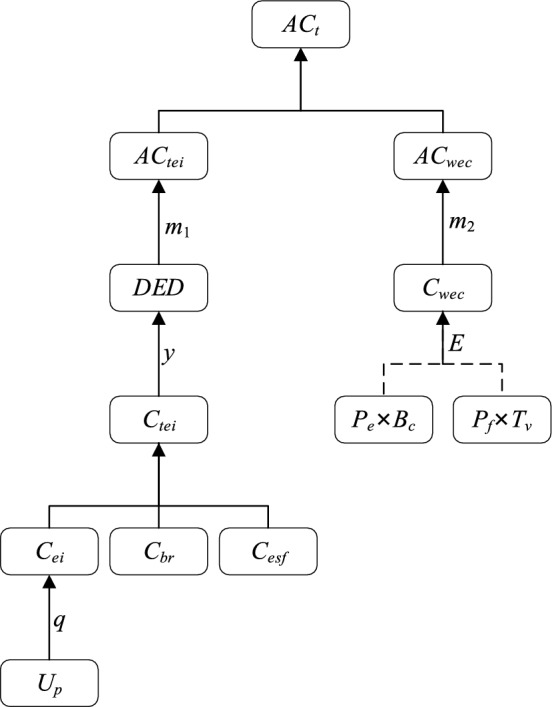
The relationship between indicators.

The relationship between indicators is shown in Fig. 4.

1. Equipment input cost (*C*_*ei*_)

*C*_*ei*_ is calculated according to the following formula:

1 $$C_{ei} = q \times U_{p}$$

where *q* refers to the quantity of equipment purchased and *U*_*p*_ refers to the unit price of equipment.

(2) Total equipment input cost (*C*_*tei*_)

Total equipment input cost includes equipment input cost (*C*_*ei*_), battery replacement cost (*C*_*br*_*)* and energy supplement facilities cost (*C*_*esf*_). Therefore, the total equipment input cost (*C*_*tei*_) can be expressed as follows:

2 $$C_{tei} = C_{ei} + \, C_{br} + C_{esf}$$

(3) Daily equipment depreciation (*DED*)

*DED* is calculated according to the following formula:

3 $$DED = C_{tei} /{ 365} \times y$$

where *y* represents the years of depreciation.

(4) Amortization of total equipment input cost (*AC*_*tei*_)

*AC*_*ei*_ is calculated according to the following formula:

4 $$AC_{tei} = DED/m_{{1}}$$

where *m*_1_ represents the daily amount of muck transfer.

(5) Working energy consumption cost (*C*_*wec*_)

*C*_*wec*_ is calculated according to the following formulas:

5 $$C_{wec} = P_{e} \times B_{c} /E$$

6 $$C_{wec} = P_{f} \times T_{v} /E$$

where* P*_*e*_ represents the price of electricity per kilowatt-hour; *P*_*f*_ represents the price of fuel per liter; *B*_*c*_ represents battery capacity; *T*_*v*_ represents tank volume; and *E* represents endurance.

Among them, formula 6 applies to electric equipment and formula 7 applies to diesel power equipment. According to the general situation of the project, the price of electricity per kilowatt-hour is CNY 0.85 and the price of fuel per liter is CNY 8.00.

(6) Amortization of working energy consumption cost (*AC*_*wec*_)

(*AC*_*wec*_) is calculated according to the following formulas:

7 $$AC_{wec} = C_{wec} /m_{{2}}$$

where *m*_2_ represents the amount of muck transfer per hour.

(7) Amortization of total cost (*AC*_*t*_)

The amortization of total cost (*AC*_*t*_) includes amortization of equipment input cost (*AC*_*tei*_) and amortization of working energy consumption cost (*AC*_*wec*_). Because the trial period of the equipment is not long, the maintenance cost is not considered in this analysis. Therefore, the Amortization of total cost (*AC*_*t*_) can be expressed as follows:

8 $$AC_{t} = AC_{tei} + AC_{wec}$$

### Excavator

Table 4 meticulously unfolds a comparative analysis between the electric excavator (XE215E) and its diesel-powered counterpart, each embodying distinct operational and economic characteristics that warrant an in-depth exploration within the context of tunnel construction.

**Table 4:** Comparison between electric excavator and diesel power products of the same class.

Contrast item		Unit	Electric excavator(XE215E)	Diesel power excavator (the same class as electric excavator)
Equipment parameters	Length	mm	10,050	9625
	Width	mm	2990	2990
	Height	mm	3100	3100
	Maximum mechanical output power of engine	kW	215	135
	Bucket digging force	kN	149	149
	Bucket capacity	m^3^	1	1
Equipment purchase cost	Unit price	CNY	1,550,000	1,000,000
	Quantity	/	1	1
	Equipment input cost	CNY	1,550,000	1,000,000
	Energy supplement facilities cost	CNY	120,000	10,000
	Battery replacement cost	CNY	500,000	0
	Total equipment input cost	CNY	2,170,000	1,010,000
	Daily equipment depreciation	CNY	594.52	276.71
	Amortization of total equipment input cost	CNY/m^3^	1.52	0.71
Working energy consumption cost	Battery capacity or Tank volume	kWh or L	350	400
	Energy-supply time	min	90	10
	Endurance	h	6	20
	Average energy consumption rate	kWh/h or L/h	58.33	20
	Unit price of energy consumption	CNY/kWh or CNY/L	0.85	8
	Working energy consumption cost	CNY/h	49.58	160
	Amortization of working energy consumption cost	CNY/m^3^	0.57	1.85
Amortization of total cost		CNY/m^3^	2.10	2.56

#### Equipment parameters

The electric excavator, while showcasing a formidable maximum mechanical output power of 215 kW, significantly eclipses the 135 kW of its diesel equivalent, thereby ensuring a potent power delivery without compromising on the intrinsic excavation capabilities, as both models exhibit an identical bucket digging force of 149 kN. However, the electric excavator, with a length of 10,050 mm, surpasses its diesel counterpart by 425 mm, potentially introducing navigational complexities within the restricted confines of tunnel environments, thereby necessitating strategic operational planning to safeguard both efficiency and safety. Moreover, the endurance time of the electric excavator, approximately one-third of the diesel model, demands frequent and strategically planned charging sessions to mitigate potential workflow disruptions.

#### Economic considerations

While the electric excavator necessitates a considerably higher initial investment, approximately 1.55 times that of the diesel variant, its working energy consumption cost is less than 50%, heralding potential long-term operational savings. Despite the initial financial outlay, the electric excavator, over an extended period of use, emerges as a more economically prudent option, with cost amortization approximately 18% lower than the diesel power excavator, thereby presenting a compelling economic narrative for its adoption.

#### In-depth analysis

The electric excavator, despite its frequent charging requisites, operates with zero emissions, aligning seamlessly with global sustainability initiatives and potentially offering long-term economic and environmental dividends, especially in protracted projects. The frequent charging requirements necessitate astute strategic planning to seamlessly integrate charging sessions into the operational workflow, thereby minimizing disruptions and adhering to project timelines. Furthermore, the zero-emission operation of the electric excavator enhances the safety and health conditions within the tunnel, mitigating workers' exposure to harmful emissions and contributing to a healthier, more conducive working environment.

In summation, the electric excavator, despite presenting initial adaptation and investment challenges, aligns impeccably with sustainability, long-term economic viability, and enhanced health and safety conditions, thereby substantiating its potential as a formidable alternative to traditional diesel-powered excavators in tunnel construction projects amidst the global pivot towards sustainable practices.

### Loader

Table 5 unfolds a comprehensive comparison between the electric loader (856E-MAX) and its diesel-powered equivalent (856H-MAX), each embodying distinct operational and economic characteristics that warrant a thorough exploration within the context of tunnel construction.

**Table 5:** Comparison between electric loader and diesel power products of the same class.

Contrast item		Unit	Electric loader(856E-MAX)	Diesel power loader(856H-MAX)
Equipment parameters	Length	mm	8980	8673
	Width	mm	2920	2970
	Height	mm	3500	3500
	Rated load	kg	5000	5000
	Maximum mechanical output power of engine	kW	180	168
	Maximum breakout force	kN	160	173
	Maximum tractive force	kN	176	180
	Rated bucket capacity	m^3^	3.50	3.50
	Maximum tipping load	kg	12,250	12,000
	Maximum dump height	mm	3415	3410
	Maximum dump reach	mm	1138	1138
Equipment purchase cost	Unit price	CNY	830,000	550,000
	Quantity	/	2	2
	Equipment input cost	CNY	1,660,000	1,100,000
	Energy supplement facilities cost	CNY	120,000	10,000
	Battery replacement cost	CNY	350,000	0
	Total equipment input cost	CNY	2,130,000	1,110,000
	Daily equipment depreciation	CNY	583.56	304.11
	Amortization of total equipment input cost	CNY/m^3^	1.50	0.78
Energy consumption cost	Battery capacity or Tank volume	kWh or L	360	300
	Energy-supply time	min	90	10
	Endurance	h	6	15
	Average energy consumption rate	kWh/h or L/h	60	20
	Unit price of energy consumption	CNY/kWh or CNY/L	0.85	8
	Working energy consumption cost	CNY/h	51	160
	Amortization of working energy consumption cost	CNY/m^3^	0.59	1.85
Amortization of total cost		CNY/m^3^	2.08	2.63

#### Equipment parameters

The electric loader, with a maximum mechanical output power of 180 kW, slightly outperforms the diesel-powered loader, which delivers 168 kW, thereby ensuring a potent power delivery without compromising on intrinsic loading capabilities. However, it's crucial to note that the electric loader exhibits smaller maximum breakout and tractive forces compared to its diesel counterpart, potentially influencing its performance in certain operational contexts. Additionally, the electric loader, extending to 8,980 mm, surpasses its diesel counterpart by 307 mm, potentially introducing navigational complexities within the restricted confines of tunnel environments, thereby necessitating strategic operational planning to safeguard both efficiency and safety. Moreover, the endurance time of the electric loader, approximately one-third of the diesel model, demands frequent and strategically planned charging sessions to mitigate potential workflow disruptions.

#### Economic considerations

While the electric loader demands a notably higher initial investment, approximately 1.51 times that of the diesel variant, its working energy consumption cost is less than 40%, heralding potential long-term operational savings. Despite the initial financial outlay, the electric loader, over an extended period of use, emerges as a more economically prudent option, with cost amortization approximately 20% lower than the diesel power loader, thereby presenting a compelling economic narrative for its adoption.

#### In-depth analysis

The electric loader, despite its frequent charging requisites, operates with zero emissions, aligning seamlessly with global sustainability initiatives and potentially offering long-term economic and environmental dividends, especially in protracted projects. The frequent charging requirements necessitate astute strategic planning to seamlessly integrate charging sessions into the operational workflow, thereby minimizing disruptions and adhering to project timelines. Furthermore, the zero-emission operation of the electric loader enhances the safety and health conditions within the tunnel, mitigating workers’ exposure to harmful emissions and contributing to a healthier, more conducive working environment.

In summation, the electric loader, despite presenting initial adaptation and investment challenges, aligns impeccably with sustainability, long-term economic viability, and enhanced health and safety conditions, thereby substantiating its potential as a formidable alternative to traditional diesel-powered loaders in tunnel construction projects amidst the global pivot towards sustainable practices.

### Dump truck

Table 6 provides a detailed comparison between the electric dump truck (SYM3251ZZX3BEV319) and its diesel-powered counterpart (SYM3257ZZX1E), each embodying distinct operational and economic characteristics that warrant a thorough exploration within the context of tunnel construction.

**Table 6:** Comparison between electric dump truck and diesel power products of the same class.

Contrast item		Unit	Electric dump truck (SYM3251ZZX3BEV319)	Diesel power dump truck (SYM3257ZZX1E)
Equipment parameters	Length	mm	8720	9200
	Width	mm	2540	2540
	Height	mm	3520	3350
	Maximum mechanical output power of engine	kW	400	360
	Curb weight	t	16.80	12.43
	Maximum allowable total mass	t	25	25
	Driving form	/	6 × 4	6 × 4
Equipment purchase cost	Unit price	CNY	750,000	400,000
	Quantity	/	8	6
	Equipment input cost	CNY	6,000,000	2,400,000
	Energy supplement facilities cost	CNY	240,000	20,000
	Battery replacement cost	CNY	0	0
	Total equipment input cost	CNY	6,240,000	2,420,000
	Daily equipment depreciation	CNY	854.79	331.51
	Amortization of total equipment input cost	CNY/m^3^	2.19	0.85
Working energy consumption cost	Battery capacity or Tank volume	kWh or L	350	400
	Energy-supply time	min	90	10
	Endurance	h	8	25
	Average energy consumption rate	kWh/h or L/h	43.75	16
	Unit price of energy consumption	CNY/kWh or CNY/L	0.85	8
	Working energy consumption cost	CNY/h	37.19	128
	Amortization of working energy consumption cost	CNY/m^3^	0.43	1.48
Amortization of total cost		CNY/m^3^	2.62	2.33

#### Equipment parameters

Power Dynamics: The electric dump truck, with a maximum mechanical output power of 400 kW, surpasses the 360 kW offered by the diesel-powered dump truck, ensuring a robust power delivery while navigating through the tunnel environments.

Weight and Load Capacity: The electric dump truck, with a curb weight of 16.80 t, is notably heavier than its diesel counterpart, which weighs 12.43 t. This additional weight, attributed to the batteries and other components, reduces the load capacity of the electric dump truck by approximately 4 tons (or about 2 cubic meters) of tunnel dregs per trip under the maximum allowable total mass constraint. Consequently, to achieve equivalent workload, the number of electric dump trucks needs to be augmented by 25%.

#### Economic considerations

Initial Investment and Operational Costs: The electric dump truck demands a significantly higher initial investment, approximately 2.5 times that of the diesel variant, especially considering the need to increase the number of vehicles to achieve the same workload. Despite the electric dump truck presenting lower working energy consumption costs, the elevated initial investment and increased vehicle quantity result in a total cost amortization that is higher than the diesel power truck.

#### In-depth analysis

Environmental and Operational Efficiency: The electric dump truck, while operating with zero emissions and aligning with global sustainability initiatives, demands strategic operational planning to ensure that the reduced load capacity and increased vehicle requirements do not adversely impact the project timelines and workflow.

Strategic Planning: The reduced load capacity of the electric dump truck necessitates astute strategic planning to seamlessly integrate the increased vehicle requirements into the operational workflow, thereby minimizing disruptions and adhering to project timelines.

Long-term Economic Viability: The higher initial investment and operational complexities of the electric dump truck, when considered in the context of long-term projects, can potentially offset the initial investment, presenting a compelling case for its adoption in tunnel construction projects, especially considering the global shift towards sustainable practices.

In conclusion, the electric dump truck, while presenting initial challenges in terms of adaptation, investment, and operational planning, aligns impeccably with sustainability and presents a potential alternative to traditional diesel-powered dump trucks in tunnel construction projects amidst the global pivot towards sustainable practices. However, its adoption necessitates meticulous planning and strategic considerations to ensure that the operational and economic implications are navigated effectively to harness its potential benefits.

In summary, the exploration and analysis of electric equipment in the context of tunnel construction, especially within the challenging environments of plateau tunnels, reveal a nuanced tapestry of economic, environmental, and health-related considerations. The preceding sections, 4.1 through 4.4, have meticulously dissected the comparative attributes of electric and diesel-powered equipment, shedding light on their respective advantages and limitations in both operational and economic spectrums.

While the initial financial outlay for electric equipment is notably higher, attributed to the elevated equipment input costs and the financial commitment to energy replenishment infrastructures, it is imperative to recognize the long-term economic and non-economic benefits that unfold. The diminished working energy consumption costs and the significant carbon reduction impact of electric equipment underscore a commitment to sustainability and environmental stewardship, which is becoming increasingly pivotal in contemporary construction practices.

Moreover, the deployment of electric equipment heralds a marked improvement in the tunnel’s air quality by mitigating exhaust emissions from fuel equipment. This not only alleviates the operational necessity and associated costs of tunnel ventilation but also crafts a healthier and safer working environment for construction personnel. The safeguarding of worker health, while not directly quantifiable in a financial matrix, is of paramount importance and presents an ethical and regulatory consideration that is integral to sustainable construction practices.

The trials involving electric muck transfer equipment, as detailed in this chapter, have not only substantiated an enhancement in the working environment and operational efficiencies within the plateau tunnel construction but have also demonstrated a tangible alignment with energy-saving and emission reduction objectives. Thus, while the economic investment in electric equipment is palpable, the amalgamation of environmental, health, and potential long-term operational benefits presents a compelling narrative for its adoption. This exploration, therefore, transcends its immediate context, offering valuable insights and reference points for future projects that seek to navigate the intricate balance between economic viability, environmental sustainability, and occupational health in tunnel construction within challenging terrains.

## Conclusions and perspectives

### Research summary and limitations

The exploration into the application of electric equipment in plateau railway tunnel projects has unveiled a myriad of insights and potential pathways towards more sustainable, efficient, and environmentally responsible construction practices. The research underscored the pivotal role of electric equipment in mitigating the multifaceted challenges posed by conventional fuel-powered machinery, particularly in the context of plateau environments characterized by reduced oxygen levels, extreme temperatures, and sensitive ecological conditions.

Through a detailed examination of various policies, technological advancements, and practical applications, the research illuminated the potential of electric equipment to not only align with China's 'Dual Carbon' targets but also to navigate through the operational, environmental, and socio-economic challenges inherent in plateau railway tunnel construction. The adoption of electric equipment, particularly in muck transfer operations, was identified as a crucial strategy to reduce pollution, enhance worker safety and health, and improve operational efficiency amidst the challenging plateau conditions.

The implications of this research extend across multiple dimensions. For the research community, this study contributes to the burgeoning field of sustainable construction practices by providing empirical evidence on the efficacy of electric equipment in challenging environments. It lays the groundwork for further exploration into alternative energy solutions within the construction industry. For practice, the findings offer actionable insights for construction project managers and policymakers looking to reduce environmental impact and enhance efficiency in plateau railway tunnel projects. The adoption of electric muck transfer equipment, as demonstrated in our study, provides a tangible pathway towards achieving China's 'Dual Carbon' goals within the construction sector. For society, this research underscores the potential for significant environmental benefits, including reduced greenhouse gas emissions and lessened ecological disturbance, contributing to a more sustainable future.

While the research provides a comprehensive overview and analysis of the current situation, several limitations must be acknowledged:

## Technological maturity：

The research is constrained by the current technological maturity and availability of electric construction machinery and equipment. The rapidly evolving nature of technology may introduce new solutions and challenges that are not addressed in the present study.

## Data availability

The availability and accessibility of data, particularly pertaining to the real-world application, performance, and challenges of electric equipment in various tunneling projects, are limited. This restricts the depth and breadth of the analysis and findings.

Diverse Working Conditions: The research primarily focuses on the application of electric equipment in plateau railway tunnel projects, which may not fully encapsulate the diverse range of working conditions and challenges encountered in different geographical and environmental contexts.

Economic and Policy Dynamics: The economic and policy dynamics are subject to change, influenced by various factors like technological advancements, market conditions, and governmental policies, which might impact the feasibility and applicability of the findings in future contexts.

The aforementioned limitations necessitate a cautious and adaptive approach to applying the findings and recommendations of the research in practical scenarios. Future research and applications should consider the evolving technological landscape, emerging data, and changing economic and policy dynamics to ensure the relevance and efficacy of strategies and solutions in real-world contexts.

In light of these limitations, the findings and recommendations of the research serve as a foundational framework, providing valuable insights and directions for further exploration, development, and application of electric equipment in plateau railway tunnel projects. The continuous evolution of technologies, methodologies, and policies will invariably introduce new opportunities and challenges, warranting ongoing research and dialogue among stakeholders to navigate towards more sustainable, efficient, and responsible construction practices.

### Policy recommendations and practical guidelines

Navigating through the complexities of adopting electric equipment in plateau railway tunnel projects necessitates a robust framework of policy recommendations and practical guidelines, wherein governmental support emerges as a linchpin, providing financial and infrastructural backing through policies, incentive programs, and a stringent regulatory framework that champions the use of environmentally congenial technologies in construction ventures, particularly within the ecologically delicate plateaus. In addition to the immediate operational and environmental benefits, the implementation of these policy recommendations and practical guidelines has profound implications for research, practice, and society. By fostering an ecosystem that encourages the use of electric equipment, we not only advance the research agenda in sustainable construction practices but also set a precedent for industry standards that prioritize environmental stewardship and worker safety. These guidelines serve as a model for other sectors, demonstrating the tangible benefits of integrating sustainable technologies into practical applications. On a societal level, these practices contribute to the realization of a greener, more sustainable future, aligning construction practices with global sustainability goals and enhancing public health through reduced emissions and pollution. The meticulous formulation and rigorous enforcement of standards for electric equipment become imperative, ensuring a steadfast adherence to safety, reliability, and efficiency across a spectrum of operational contexts, while on a practical note, the crafting of a phased strategy for the adoption of electric equipment, which prioritizes the foundational establishment of charging and battery-swap infrastructure, coupled with the implementation of comprehensive training programs for operators and maintenance personnel, becomes crucial to seamlessly navigate the operational and logistical challenges intertwined with the integration of novel technologies into extant workflows. Concurrently, the establishment of risk management protocols, designed to adeptly address potential challenges and disruptions related to the utilization of electric equipment in tunnel construction, acts as a safeguard, fortifying against operational and financial adversities.

### Future research directions

Envisioning the future research directions in the domain of electric equipment utilization in plateau railway tunnel projects encompasses a multifaceted exploration into technological advancements, operational efficacy, socio-economic implications, and interdisciplinary research, with a pronounced focus on enhancing battery technology, delving into research that seeks to elevate energy density, expedite charging capabilities, and augment longevity, while also exploring the integration of automation and AI technologies to enhance efficiency and safety in tunnel construction activities. Beyond technological advancements, future research must emphasize the socio-economic and environmental impacts of electric equipment adoption. This includes studying the effects on worker health, community well-being, and contributing to sustainable development goals. Interdisciplinary research that bridges technology with policy analysis, social sciences, and environmental studies will be crucial for understanding the full spectrum of impacts and opportunities presented by electric equipment in construction. Investigative pursuits into innovative materials and technologies that can bolster the durability and performance of electric equipment, especially within the harsh environments of plateaus, emerge as a pivotal research avenue, while operational dynamics, environmental impact studies, and health and safety investigations will pave the way for data-driven insights and guidelines that optimize and safeguard tunnel construction practices. Furthermore, comprehensive economic analyses, social impact explorations, and investigations into the supply chain implications will illuminate the broader socio-economic ramifications and strategic considerations related to the adoption of electric equipment, and fostering intersectoral collaboration, engaging in international research collaborations, and exploring the broader sustainability implications of adopting electric equipment will ensure that practices are globally informed, technologically advanced, and holistically sustainable.

## Data Availability

The datasets used and analysed during the current study are available from the corresponding author on reasonable request.

## List of symbols

*C*_*ei*_: Equipment input cost
*C*_*br*_: Battery replacement cost
*C*_*esf*_: Energy supplement facilities cost
*C*_*tei*_: Total equipment input cost
*C*_*wec*_: Working energy consumption cost
*DED*: Daily equipment depreciation
*AC*_*t*__*ei*_: Amortization of equipment input cost
*AC*_*wec*_: Amortization of working energy consumption cost
*AC*_*t*_: Amortization of total cost

## Symbols

*q*: Quantity of equipment purchased
*y*: Years of depreciation
*m*_1_: Daily amount of muck transfer
*m*_2_: Amount of muck transfer per hour
*P*_*e*_: Price of electricity per kilowatt-hour
*P*_*f*_: Price of fuel per liter
*B*_*c*_: Battery capacity
*T*_*v*_: Tank volume
*E*: Endurance
