# Author Correction: Application and configuration analysis of electric muck transfer equipment in plateau railway tunnel: a case study in southwest China

**DOI:** 10.1038/s41598-024-59431-7

**Published:** 2024-04-15

**Authors:** Xiaoxu Yang, Yuming Liu, Kai Liu, Jianying Wei, Guangzhong Hu, Shifan Pei

**Affiliations:** School of Economics and Management, Beijing Jiaotong University, Beijing, 100044 China

Correction to: *Scientific Reports* 10.1038/s41598-024-57628-4, published online 27 March 2024

In the original version of this Article, Jianying Wei was incorrectly affiliated with ‘Department of Systems Engineering, City University of Hong Kong, Hong Kong, 999077, China’. The correct Affiliation is listed below:

‘School of Economics and Management, Beijing Jiaotong University, Beijing 100044, China.’

The original Article has been corrected.

